# Citrus peel essential oil nanoformulations to control the tomato borer, *Tuta absoluta*: chemical properties and biological activity

**DOI:** 10.1038/s41598-017-13413-0

**Published:** 2017-10-12

**Authors:** Orlando Campolo, Asma Cherif, Michele Ricupero, Gaetano Siscaro, Kaouthar Grissa-Lebdi, Agatino Russo, Lorena M. Cucci, Patrizia Di Pietro, Cristina Satriano, Nicolas Desneux, Antonio Biondi, Lucia Zappalà, Vincenzo Palmeri

**Affiliations:** 1 University of Reggio Calabria, Dipartimento di AGRARIA, Loc. Feo di Vito, 89122 Reggio Calabria, Italy; 2 University of Catania, Department of Agriculture, Food and Environment, via Santa Sofia 100, 95123 Catania, Italy; 3 University of Carthage, Laboratoire d’Entomologie-Acarologie, Institut National Agronomique de Tunisie, 43 Avenue Charles Nicolle, 1082 Cité Mahrajène, Tunis Tunisia; 4 University of Catania, Department of Chemical Sciences, Viale Andrea Doria 6, 95125 Catania, Italy; 5 INRA (French National Institute for Agricultural Research), Université Nice Sophia Antipolis, CNRS, UMR 1355-7254, Institut Sophia Agrobiotech, 06903 Sophia Antipolis, France

## Abstract

The repeated use of conventional synthetic pesticides in crop protection leads to resistance development by pests along with a negative impact on the environment, particularly non-target arthropods. Plant-derived active compounds, such as essential oils (EOs), play a key role in sustainably controlling pests. The lethal and sublethal activity of citrus peel EOs as emulsions and included in polyethylene glycol (PEG) nanoparticles (EO-NPs) was determined against the invasive tomato pest *Tuta absoluta*. Their effects on the plants were also assessed. The results showed an overall good insecticidal activity of the compounds tested, with a higher mortality through contact on eggs and larvae by EO emulsions and through ingestion on larvae by EO-NPs. The nanoformulation also significantly reduced the visible toxic effects on the plants. The data collected suggest that these natural compounds, especially when nanoformulated, could be successfully used in integrated pest management programs for *T. absoluta*.

## Introduction

The tomato crop has a very high economical and social significance worldwide and has recently been threatened by an invasive pest, the South American tomato pinworm or tomato borer *Tuta absoluta* (Meyrick) (Lepidoptera: Gelechiidae)^1^. This moth has a high reproductive potential, completing up to 13 generations per year^2^. The larvae feed inside the tomato leaves, stems and fruits, leading to severe yield loss in greenhouse and open-field tomato crops^1^. The potential damage and high growth rate of this pest have pushed growers to increase the number of insecticide applications throughout the tomato production cycle^1^. This has led to the rapid development of insecticide resistance^3,4^ and has also had a considerable negative impact on non-target organisms, such as natural enemies and pollinators^5–7^. As a consequence, alternative control tools to conventional synthetic pesticides have been tested and/or implemented within tomato Integrated Pest Management (IPM) packages. The efficacy of resistant tomato varieties^8^, synthetic pheromones^9^, mineral deterrents^10^, ecological services provided by fortuitous natural enemies^11^, with particular reference to generalist Heteroptera predators^12–14^, have all been tested with contrasting results.

In terms of sustainable control tools, plant-derived active compounds, i.e. botanical insecticides, have historically occupied a key role^15^, but recently research has increased in the development of new compounds and/or the inclusion in IPM packages of old ones, e.g. neem essential oils^16,17^. The idea supporting the use of such substances derives from the evolution of natural plant defence mechanisms, for which plants have developed an array of secondary metabolites used to protect themselves against herbivores and pathogens^18^. The route of exposure, the environmental conditions and the physiological status of the target organism can affect the toxicity of botanicals^19–21^. These substances have then been tested against a large number of arthropod pests, such as ingestion larvicides^22^, contacticides^23^, fumigants^24^, repellents^25,26^ and antifeedants^27^. Among the various extracts, plant essential oils (EOs) have shown a good potential in controlling insect pests, as well as managing bacterial and fungal plant pathogens^28–30^.

Despite their promising properties, EO-based insecticides have some drawbacks (e.g. volatility, poor water solubility, environmental degradation) related to their chemical composition, which can negatively affect their application^31^. The encapsulation of EOs inside nanoparticles could reduce these problems, improving at the same time the efficacy and the induction of systemic activity due to the small size of the particles^32,33^. Specifically, polyethylene glycol (PEG) functionalised nanoparticles (NPs) considerably improve solubility in water and control pesticide release^34,35^.

The aim of this study was thus to assess the insecticidal activity of different citrus EOs against eggs and larvae of the tomato borer *T. absoluta*. Both emulsions and PEG nanoparticles containing EOs were evaluated through contact and exposure ingestion route in order to determine their lethal and sublethal effects. The potential toxic effects on tomato plants were also investigated.

## Results

### EO-NPs characterization

**Figure 1:**
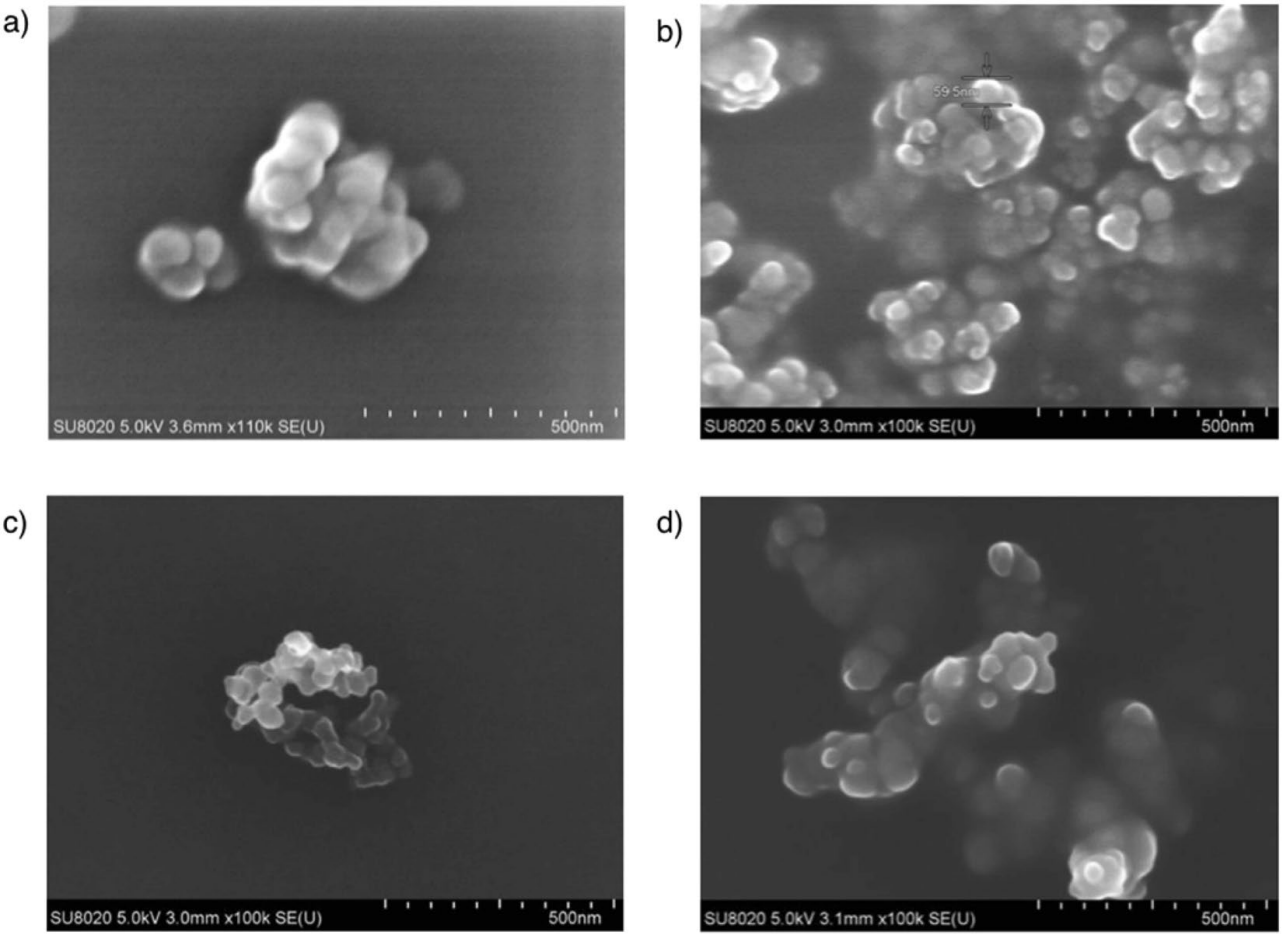
SEM micrographs of unloaded and EO-loaded NPs. (**a**) Bare PEG; (**b**) Lemon EO-NP, (**c**) Mandarin EO-NP, (**d**) Sweet orange EO-NP.

Lemon EO-NPs showed a loading efficiency of 96% (w/w), as determined spectrophotometrically (see Figures S1, S2 and Table S1 in Supplementary Material), and an average size of approximately 240 ± 2.51 nm. Both mandarin and sweet orange EO-NPs showed a smaller mean diameter, of about 212 ± 0.04 nm and 216 ± 0.63 nm respectively, and a loading efficiency comparable to that of lemon EO-NP, i.e. 92% (w/w) mandarin-NP and 88% (w/w) orange EO-NP. The relatively low values of the polydispersity index (0.23–0.34) for the EO-loaded NPs (Table 1) indicated the homogeneity of the formulations. Moreover, the SEM observations confirmed that the size of all EO-NPs falls within the sub-micrometer range (Fig. 1), each NP consisting of clusters of spherical features approximately 50 nm of size. Finally, all the EO-loaded NPs exhibited a negative surface charge of about −30 mV, whereas the unloaded nanoparticles were neutral (Table 1).

**Table 1:** Average size (mean value ± SE), polydispersity index (PDI), surface charge and loading determined for the various EO- PEG NPs dissolved in ethanol/water (3:1, v/v).

Sample	d (nm)±SE	PDI	Zeta potential (mV)	EO loading (w/v)	Loading efficiency (w/w)
Bare PEG-NP	53 ± 2	0.2	0.27 (±0.03)	—	—
Lemon EO-NP	240 ± 2.51	0.34	−30.60 (±0.58)	24%	96%
Mandarin EO-NP	212.05 ± 0.04	0.26	−31.13 (±0.38)	23%	92%
Sweet Orange EO-NP	216.6 ± 0.63	0.23	−27.80(±0.57)	22%	88%

### Toxicological bioassays

Repeated-measures analysis showed that both the application rate (*F* = 104.249; df = 1; *p* < 0.001) and the formulation (*F* = 5.532; df = 1; *p* < 0.001), i.e., EOs or EO-NPs, had a significant effect on the mortality registered 24 h, 72 h after the treatment, and at adulthood.

### Contact Toxicity on Eggs (CTE)

The mortality of eggs sprayed with indoxacarb (positive control) was 0.50%, 4.5% and 100% after 24, 72 h, and at adulthood, respectively. Eggs, exposed to contact toxicity, were less susceptible to both tested EO formulations than larvae exposed through the translaminar and ingestion route (*F* = 203.375; df = 2;  *p*< 0.001). Eggs that died during the trial ranged from 0 to 12%, without significant differences compared to the water or the TWEEN control treatments (*F* = 1.680; df = 1; *p* = 0.178). Overall, the egg mortality was mainly influenced by the application rate (*F* = 24.534; df = 4; *p* < 0.001), whereas no significant differences were highlighted in the mortality registered comparing the formulation (i.e., EO emulsion and EO-NP: *F* = 1.628; df = 1; *p* = 0.203) and the different EOs (*F* = 1.468; df = 2; *p* < 0.232). After 24 hours (Table 2) and at the maximum application rate, the highest mortality was achieved in the sweet orange EO emulsion treatment. In the second sampling (72 h after the treatment), the maximum mortality was registered in the sweet orange EO-NP treatment at the maximum application rate (40 mg × mL^−1^). A similar trend was observed when analysing the proportion of emerged adults (Table 2).

**Table 2:** Mean mortality (percentage ± SE) of *T. absoluta* eggs exposed to different EO and EO-NP formulations at the various application rates in the contact toxicity on eggs (CTE) bioassay.

Time	Application Rate (mg × mL^−1^)	Lemon		Mandarin		Sweet Orange	
		EO	EO-NP	EO	EO-NP	EO	EO-NP
24 h	2.5	0 ± 0	0 ± 0	0 ± 0	0 ± 0	0 ± 0	0 ± 0
	5.0	0 ± 0	0 ± 0	0 ± 0	0 ± 0	0 ± 0	0 ± 0
	10.0	0 ± 0	0 ± 0	0 ± 0	0 ± 0	0 ± 0	0 ± 0
	20.0	0 ± 0	0 ± 0	0 ± 0	0 ± 0	0 ± 0	0 ± 0
	40.0	0 ± 0b	0 ± 0b	0 ± 0b	0 ± 0b	8 ± 3.7a	2 ± 2ab
72 h	2.5	0 ± 0b	0 ± 0b	0 ± 0b	2 ± 2ab	6 ± 2.4a	0 ± 0b
	5.0	0 ± 0c	6 ± 4ab	10 ± 3.2a	8 ± 2a	6 ± 2.4ab	0 ± 0c
	10.0	8 ± 4.9a	8 ± 4.9a	12 ± 5.8a	8 ± 3.7a	8 ± 2a	6 ± 2.4a
	20.0	10 ± 3.2a	10 ± 3.2a	18 ± 5.8a	14 ± 5.1a	8 ± 3.7a	14 ± 2.4a
	40.0	12 ± 4.9b	12 ± 2b	22 ± 3.7ab	12 ± 5.8b	22 ± 3.7ab	40 ± 4.5a
to adult	2.5	8 ± 2a	6 ± 4ab	2 ± 2ab	2 ± 2ab	6 ± 2.4ab	0 ± 0a
	5.0	12 ± 4.9a	8 ± 2a	10 ± 3.2a	8 ± 3.7a	10 ± 3.2a	2 ± 2a
	10.0	16 ± 4a	12 ± 4.9a	16 ± 5.1a	10 ± 4.5a	14 ± 5.1a	14 ± 2.4a
	20.0	16 ± 2.4a	16 ± 4a	26 ± 4a	16 ± 5.1a	14 ± 4a	20 ± 0a
	40.0	22 ± 2b	18 ± 3.7b	26 ± 2.4b	26 ± 4b	24 ± 2.4b	46 ± 5.1a

Different letters within the same row indicate statistical differences at *p* < 0.05 (GLM, Duncan’s multiple range test). Statistics are based on transformed data.

### Translaminar Toxicity on Larvae (TTL)

In the two control treatments, no larvae died during the first 72 h after the treatment, whereas 0% and 4% did not reach the adult stage for water and TWEEN, respectively (*F* = 0; df = 1; *p* = 1). In the first sampling (24 h after the treatment), sweet orange EO emulsion was the most effective in killing the moth’s larvae. In all cases, the EO emulsions were, on average more effective than the EO-NPs (*F* = 49.568; df = 1; *p* < 0.001) (Table 3). In the second sampling (72 h), the mortality of *T. absoluta* larvae increased in all the treatments, and in the sweet orange EO-NP, the mortality rates almost doubled compared to the first sampling. The number of adults that emerged after the EO application, was significantly higher in the EO-NP treated larvae compared to the EO emulsion. The only exception was the Lemon EO-NP treatment, where the emergence reduction was higher (12%) compared to the respective EO emulsion formulation (*F* = 15.541; df = 1; *p* < 0.001). Indoxacarb killed 60 ± 3.16, 70 ± 9.35 and 92 ± 3.74% of the exposed larvae after 24, 72 h, and at adult emergence, respectively.

**Table 3:** Mean mortality (percentage ± SE) of *T. absoluta* larvae exposed to different EO and EO-NP formulations at the different application rates in the translaminar toxicity on larvae (TTL) bioassay.

Time	Application Rate (mg × mL^−1^)	Lemon		Mandarin		Sweet Orange	
		EO	EO-NP	EO	EO-NP	EO	EO-NP
24 h	2.5	10 ± 3.2a	16 ± 4a	14 ± 5.1a	16 ± 4	20 ± 4.5a	16 ± 4a
	5.0	16 ± 4bc	16 ± 4c	30 ± 4.5ab	20 ± 3.2abc	32 ± 2a	16 ± 4bc
	10.0	20 ± 0b	22 ± 2b	32 ± 3.7a	18 ± 3.7b	34 ± 2.4a	22 ± 3.7b
	20.0	50 ± 5.5b	32 ± 4.9c	70 ± 3.2a	38 ± 5.8bc	74 ± 4a	32 ± 4.9c
	40.0	58 ± 3.7c	48 ± 4.9c	76 ± 2.4b	50 ± 4.5	90 ± 5.5a	64 ± 4bc
72 h	2.5	30 ± 3.2a	24 ± 7.5a	36 ± 4a	24 ± 6a	36 ± 2.4a	22 ± 3.7a
	5.0	40 ± 8.9ab	24 ± 7.5b	44 ± 4a	30 ± 4.5ab	38 ± 3.7ab	24 ± 5.1c
	10.0	42 ± 6.6ab	30 ± 3.2b	46 ± 4a	36 ± 4ab	48 ± 3.7a	32 ± 2b
	20.0	64 ± 7.5a	36 ± 7.5b	76 ± 5.1a	42 ± 3.7b	76 ± 2.4a	40 ± 4.5b
	40.0	66 ± 2.4ab	52 ± 4.9b	78 ± 3.7b	62 ± 3.7b	92 ± 3.7a	80 ± 4.5b
to adult	2.5	38 ± 6.6a	28 ± 10.2a	36 ± 7.5a	26 ± 4a	38 ± 3.7a	24 ± 4a
	5.0	46 ± 6a	30 ± 7.1b	44 ± 4ab	30 ± 3.2ab	40 ± 4.5ab	28 ± 3.7b
	10.0	48 ± 3.7a	44 ± 2.4ab	46 ± 2.4a	36 ± 2.4b	48 ± 3.7a	36 ± 2.4b
	20.0	66 ± 6bc	56 ± 5.1cd	76 ± 4b	44 ± d	90 ± 3.2a	44 ± 4d
	40.0	70 ± 3.2bc	82 ± 3.7b	78 ± 3.7b	62 ± 5.8c	92 ± 2a	82 ± 3.7b

Different letters within the same row indicate statistical differences at *p* < 0.05 (GLM, Duncan’s multiple range test). Statistics are based on transformed data.

### Ingestion Toxicity on Larvae (ITL)

In the ITL trial, only two larvae failed to reach the adult stage in the controls. Conversely to findings in the TTL trial, the EO-NP formulations killed a higher number of larvae (*F* = 29.106; df = 1; *p* < 0.001) than the EO emulsions. Throughout the entire trial, the mandarin EO-NP formulation was the most effective against the wandering larvae (max mortality = 94 ± 4%), whereas the lemon EO emulsion was only able to kill a maximum of 38 ± 3.7% of the exposed larvae (Table 4). The mortality induced by indoxacarb ranged from 48 ± 3.74% (24 h) to 100% at the end of the trial.

**Table 4:** Mean mortality (percentage ± SE) of *T. absoluta* larvae exposed to different EO and EO-NP formulations at the different application rates in the ingestion toxicity on larvae (ITL) bioassay.

Time	Application Rate (mg × mL^−1^)	Lemon		Mandarin		Sweet Orange	
		EO	EO-NP	EO	EO-NP	EO	EO-NP
24 h	2.5	4 ± 2.4c	12 ± 3.7b	24 ± 4a	28 ± 3.7a	18 ± 2ab	26 ± 4a
	5.0	8 ± 3.7c	12 ± 3.7bc	30 ± 4.5a	36 ± 4a	20 ± 3.2ab	28 ± 3.7a
	10.0	12 ± 3.7b	16 ± 2.4b	32 ± 3.7a	38 ± 3.7a	18 ± 2b	32 ± 4.9a
	20.0	18 ± 4.9d	22 ± 2 cd	40 ± 3.2b	60 ± 4.5a	22 ± 2 cd	30 ± 3.2bc
	40.0	24 ± 2.4b	30 ± 3.2b	64 ± 5.1a	72 ± 3.7a	30 ± 4.5b	62 ± 3.7a
72 h	2.5	8 ± 2c	16 ± 2.4bc	38 ± 3.7a	44 ± 8.1a	28 ± 3.7ab	42 ± 4.9a
	5.0	12 ± 2d	20 ± 3.2c	44 ± 4a	48 ± 3.7a	34 ± 2.4b	48 ± 2a
	10.0	18 ± 2c	20 ± 3.2c	46 ± 5.1b	62 ± 3.7a	36 ± 2.4b	62 ± 3.7a
	20.0	28 ± 3.7c	38 ± 3.7c	54 ± 4b	64 ± 4ab	38 ± 2c	68 ± 3.7a
	40.0	30 ± 3.2d	40 ± 3.2cd	74 ± 6b	86 ± 4a	50 ± 3.2c	74 ± 4b
to adult	2.5	14 ± 2.4c	20 ± 3.2c	50 ± 4.5ab	58 ± 3.7a	42 ± 2b	52 ± 3.7ab
	5.0	18 ± 3.7c	22 ± 2cd	52 ± 4.9ab	64 ± 4a	46 ± 4b	60 ± 3.2a
	10.0	24 ± 2.4c	26 ± 4c	54 ± 5.1b	68 ± 4.9a	48 ± 3.7b	68 ± 3.7a
	20.0	32 ± 3.7c	46 ± 2.4b	74 ± 2.4a	72 ± 2a	50 ± 3.2b	80 ± 3.2a
	40.0	38 ± 3.7d	46 ± 2.4d	84 ± 4b	94 ± 4a	68 ± 2c	82 ± 3.7b

Different letters within the same row indicate statistical difference at *p* < 0.05 (GLM, Duncan’s multiple range test). Statistics are based on transformed data.

### Median lethal concentrations (LC_50_) for larvae

The LC_50_ values (Table 5) were not calculated for eggs because the maximum mortality registered was less than 50%. The dose-mortality data in larvae exposed to the EO formulations showed low χ^2^ and high α-values (<21.53 and >0.36, respectively). These parameters indicate the suitability of the model to estimate the LC_50_^36^. In the TTL trial, the LC_50_ values calculated for the EO-NP formulations were higher than the EO emulsion values, regardless of the essential oil used. Sweet orange EO emulsion showed the highest capacity to kill the exposed larvae. The mandarin EO-NP formulation needed the highest concentration (23.10 mg × mL^−1^) to kill 50% of the exposed larvae. Conversely, in the IL trial, the EO-NP formulations required a lower application rate than the EO emulsion formulation (Table 5).

**Table 5:** Estimated median lethal concentrations (LC_50_) of the various EOs and formulations on *T. absoluta* larvae in the translaminar toxicity on larvae (TTL) and ingestion toxicity on larvae (ITL) bioassays.

Route of exposure	Essential oil	Formulation	LC_50_ (mg × mL^−1^)	95% Fiducial limits	Slope ± SE	Intercept ± SE	χ^2^ (df = 23)
Trasnlaminar (TTL)	Lemon	EO	7.58a	3.60–13.01	0.70 ± 0.192	−0.62 ± 0.21	14.26 ns
	Lemon	EO-NP	11.06ab	8.02–15.62	1.19 ± 0.20	−1.25 ± 0.22	24.63 ns
	Mandarin	EO	6.45a	3.48–9.90	0,86 ± 0.19	−0.69 ± 0.21	21.53 ns
	Mandarin	EO-NP	23.09b	13.94–64.57	0.76 ± 0.19	−1.03 ± 0.21	10.16 ns
	Sweet Orange	EO	5.77a	4.19–7.46	1.53 ± 0.21	−1.17 ± 0.21	20.27 ns
	Sweet Orange	EO-NP	14.68b	11.18–20.48	1.11 ± 0.20	−1.31 ± 0.22	19.43 ns
Ingestion (ITL)	Lemon	EO	111.04c	41.36–4,817	0.66 ± 0.20	−1.36 ± 0.23	6.05 ns
	Lemon	EO-NP	47.4c	31.15–95.39	0.72 ± 0.20	−1.21 ± 0.22	6.214 ns
	Mandarin	EO	3.79a	2.01–5.55	0.77 ± 0.19	−0.42 ± 0.20	13.97 ns
	Mandarin	EO-NP	0.99a	0.05–2.82	0.57 ± 0.20	0.02 ± 0.21	21.31 ns
	Sweet Orange	EO	8.9b	1.46–33.71	0.43 ± 0.18	−0.41 ± 0.20	6.17 ns
	Sweet Orange	EO-NP	1.53a	0.053–3.62	0.61 ± 0.19	−0.11 ± 0.20	11.69 ns

Different letters within the same column of each trial indicate statistical differences (p < 0.05); ns = not significant (α > 0.05).

### Phytotoxicity assessment

**Figure 2:**
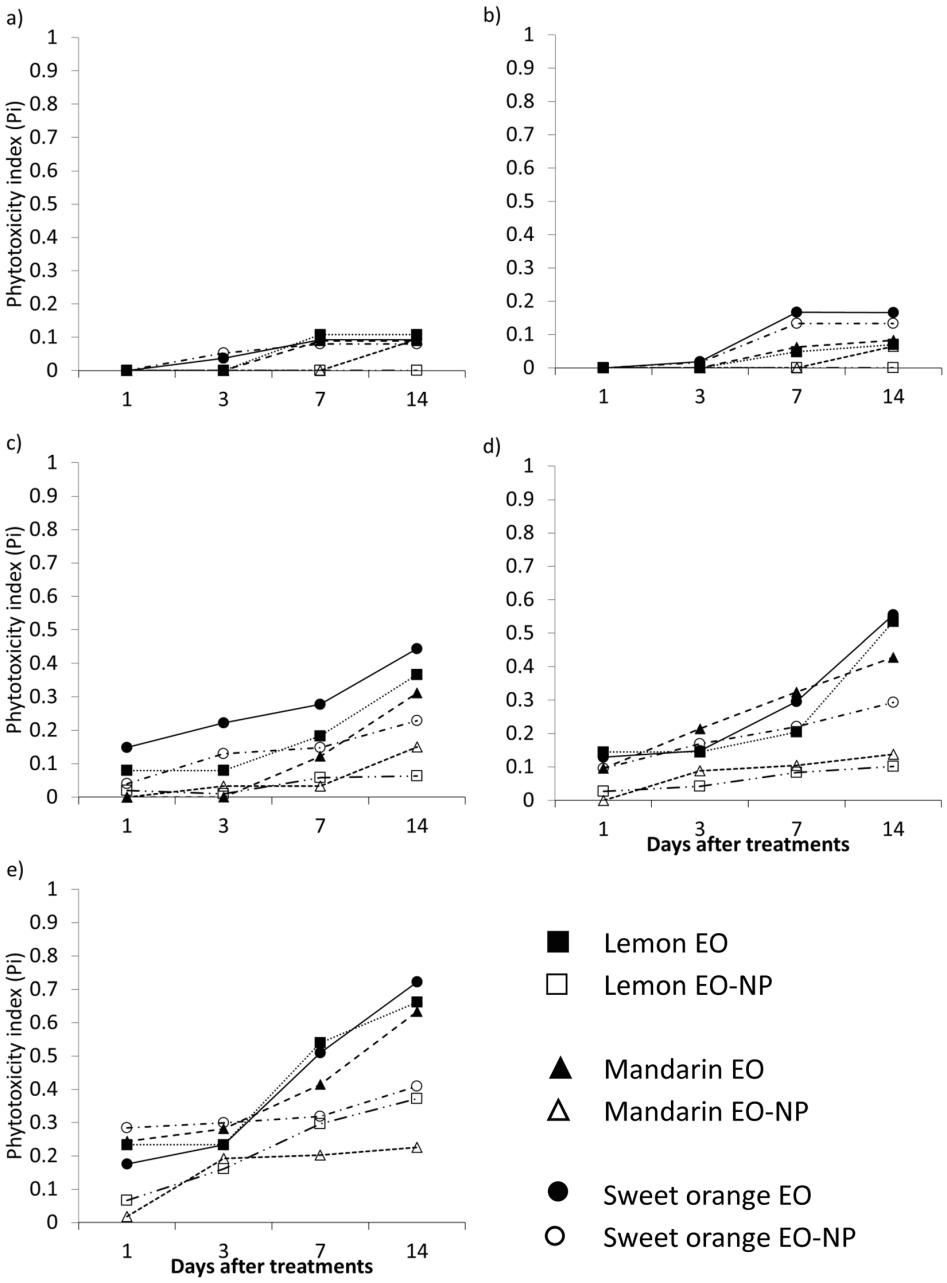
Mean values of the phytotoxicity index (P_i_) of the tested EOs and formulations recorded at 1, 3, 7, 14 days after the treatments. (**a**) 2.5 mg × mL^−1^; (**b**) 5.0 mg × mL^−1^; (**c**) 10.0 mg × mL^−1^; (**d**) 20.0 mg × mL^−1^; (**e**) 40.0 mg × mL^−1^.

The toxic effects of the tested formulations on the plants, expressed as the phytotoxicity index (P_*i*_), are shown in Fig. 2 (see Equation (1) in Data analysis section). No phytotoxicity was registered either in the water treatment or in the positive control (i.e., indoxacarb), whereas in the TWEEN 80 control, a slight phytotoxic effect (P_*i*_ = 0.09) was shown at the maximum application rate, 14d after the treatment (data not shown). The tested EOs had toxic effects on the plants which significantly depended on the formulation (i.e. EO emulsion *vs*. EO-NP, *F* = 107.69 df = 2 *p* < 0.001), the application rates and the time elapsed after the treatment (model: *F* = 73.083; df = 8; *p* < 0.001). The P_*i*_ ranged from 0 (no damage) to 0.78 registered after 14d in the plants treated with the sweet orange EO emulsion at 40 mg × mL^−1^. Among the EO-NP formulations, the sweet orange essential oil also had the highest negative impact on the plants (max P_*i*_ = 0.48). The other two EO-NPs reached their maximum P_*i*_ (0.27 and 0.21 for mandarin and lemon EO-NP, respectively) at the highest application rate and 14 days after the treatment. In all the treatments, the new vegetation grown in the 14 days after the beginning of the trial, did not show any phytotoxic effect.

## Discussion

Nanoparticles improve both the stability and effectiveness of botanical insecticides. In fact, nanoformulations can solve problems related to EO volatility, poor water solubility, and the tendency to oxidize^34^. In addition, nanoparticles are able to release the active compounds at the site of action gradually^37^, and also minimize the toxic effects on non-target organisms^38,39^. Both the size and the polydispersion index obtained in our study are comparable with those recorded for other citrus EO-NPs (i.e. Bergamot EO-NPs)^34^. The change of the zeta potential from about zero in the case of unloaded NPs to about −30 mV for the different EO-NP formulations can be considered an indicator of the extent of EO loading in the nanoparticles, as confirmed by spectroscopic analyses that indicate an efficient loading (>90% w/w) for the different EO-NP systems. Another interesting property of the EO-loaded nanoparticles is that the relatively high values of surface charges suggest a good stability of our NP formulations. A minimum of ±30 mV of zeta potential is required for a physically stable nanosuspension solely stabilized by electrostatic repulsion^40^.

Our results highlighted the good insecticidal activity of the citrus peel essential oils against the tomato borer *T. absoluta*. The different exposure routes and target instars (i.e. CTE, TTL and ITL) together with the observations regarding phytotoxicity, contributed to a thorough understanding of the potential use of the tested EOs in the field.

Despite the large number of EOs tested as insecticides, only a few studies have focused on the tomato borer. In general, very few studies on crop-pest systems are available where both the efficacy and the impact of the insecticide treatments on the plant have been assessed. In addition, most papers tend to report the acute toxicity (or repellence) of a given EO in trials lasting less than 48 h^41^, whereas the long-lasting effects of these compounds are lacking. To the best of our knowledge, this is the first study where the long-lasting effects of EOs on a crop pest and on the plants are reported.

EOs extracted from citrus fruit have been used in many industrial applications, such as perfumery, pharmacology, food industry and fine chemistry. Citrus EOs have been tested as pesticides against a number of pests^24,29,42–45^. This interest may be due to their easy availability worldwide at a reasonable cost. Very frequently citrus EOs are by-products of the citrus juice industry, and the cold-pressing technique makes the EO extraction less expensive than other methods. The cost of the nano-formulations is influenced by the manufacturing process and by the reagents (i.e. PEG) needed. The scalability of the EO nano-encapsulation process at commercial level is easy to achieve by pesticide industry because this process is already used to produce several “new generation” insecticides.

Our study suggests that eggs are less susceptible than larvae to EO-based formulations. Less than 50% of the exposed eggs in our study did not reach adult stage when treated with the most effective formulation (sweet orange EO-NP) at the maximum application rate. Insect eggs are often considered as the most vulnerable stage, however the egg response to insecticide exposure has seldom been assessed. In addition, eggs are sometimes hard to reach with insecticide applications because of their sessile conditions in hidden places^46^. In addition, the structure of the eggs protects the developing embryos and may interfere with insecticide penetration^46,47^.

Tomé *et al*.^27^ reported a negligible ovicidal activity of the growth regulator pyriproxyfen in the tomato borer, and highlighted a delayed mortality due to a reduction in the larval activity. This also happened in our study, with eggs exposed to indoxacarb showing a delayed mortality starting three days after exposure when newly emerged larvae began feeding. Similarly, the EOs led to a delayed effect on the eggs, reducing the number of emerged larvae that reached adulthood, therefore confirming their disruptive effect on insect growth^41^.

In the translaminar toxicity trial, in almost all cases the EO formulations showed a higher toxicity compared to the mortality rate in the EO-NP treatments. The EO-NPs contain a tenth part of essential oil compared to the EO emulsion, therefore, the amount of active ingredients that reached the larvae inside mines may not be enough to trigger the biological effects of NPs. By contrast, in the ingestion toxicity trial (ITL) in which larvae were transferred onto the treated leaf surface, the EO-NPs killed more larvae than the respective emulsion. Nanoparticles are known to be much more mobile than the bulk substances^48^. This characteristic enhances the penetration of the active ingredients into the insect tissue by direct contact through the insect’s cuticle or by ingestion^32,34,48^.

In our study, the insecticidal activity of the tested EOs varied with stage, exposure route, and formulation. Sweet orange EO emulsion was the most toxic against eggs (CTE) and larvae inside mines (TTL), whereas mandarin EO in the NP formulation was the most effective in the ingestion toxicity trial. In the sweet orange, mandarin, and lemon EOs, limonene was the most abundant among the compounds detected (88.7%, 59.2%, and 52.8%, respectively)^29^. The amount of limonene in citrus EOs also depends on seasonal variations, ecological, environmental and/or agronomic factors^24,29^. The insecticidal effects of limonene were confirmed in trials against stored product pests^48–53^ mealybugs and other scale insects^54^. Despite the role of single compounds in insecticidal activity^41^, the synergistic effects of complex mixtures (such as EOs) strengthen the toxicity to pests^55,56^.

Apart from their insecticidal and repellent activity, some essential oils or some of their compounds lead to negative impacts on the plants^57^. In our study, the phytotoxic effect was dose-dependent, and the EO emulsions caused more damage to the plants than the EO-NPs. Among the EOs tested, sweet orange had the strongest impact on plants, whereas mandarin was the least phytotoxic EO. In plants, most of the biological activities of EOs are mediated through direct interaction with the lipid layers of biological membranes^58^, and when used as herbicides, foliar-applied EOs caused visible damage within a few hours of their application^59^. In this study, the toxic effects on plants progressed over time, and for the first three days, the phytotoxic effects were negligible (P_i_ close to zero) even at the highest application rates.

The citrus EO-NPs were effective in controlling the target pest, while reducing the toxic effects on the plants. Further work is needed to test their efficacy under realistic field conditions. In addition, from an Integrated Pest Management (IPM) perspective, the potential lethal and sublethal effects of citrus peel essential oils and of their PEG formulations should also be assessed on non-target organisms, such as pollinators and natural enemies.

## Methods

### Insect and plant rearing

*Tuta absoluta* specimens used for all the experiments originated from infested tomato leaves collected in 2009 in organic greenhouses in south east Sicily (Italy), which were re-inoculated with adults coming from the field twice a year. The colony was maintained in the laboratory on cherry-type tomato plants (cv. Shiren). All plants used for the insect rearing, as well as those used for the experiments, were grown outdoors, under natural temperature, humidity and light conditions, in 1-liter pots, inside screened cages. No pesticides were used. Insects were reared inside polyester net cages (50 × 60 × 80 cm), in the laboratory at 24 ± 2 °C and 50 ± 10% RH. LED lamps were positioned above each cage, maintaining a photoperiod of 14:10 (L:D) according to the rearing methodology described by Zappalà *et al*.^10^.

To obtain coetaneous insect cohorts, 200 unsexed newly-emerged adults of *T. absoluta* were released inside each cage containing four potted tomato plants (height: 25 cm). The moths were left overnight to lay eggs and then removed. Eggs (72 ± 12 h old) and newly-molted second-instar larvae were used in the trials.

### Essential Oil-nanoparticle (EO-NP) preparation

Commercial citrus peel essential oils (Capua SRL, Campo Calabro Italy) of lemon (LE), mandarin (MA) and sweet orange (SO), extracted with the cold pressing technique from fruit grown in southern Italy, pesticide-free certified, were used in the trials. A total of 88 compounds were detected, with limonene being the most abundant compound (88.75, 59.19 and 52.80% for SO, MA and LE EOs respectively), Monoterpene hydrocarbons ranged from 96.08% (SO) to 91% (LE). Oxygenated compounds (aldehydes, esters and alcohols) were more abundant in LE (8.91%) than in SO (3.28%) and MA (4.36%). For complete analytical procedures and chemical characterization see Campolo *et al*.^22^.

TWEEN 80 (Polyoxyethylene (20) sorbitan monooleate) and PEG 6000 (Polyethylene glycol, molecular weight 6,000) were purchased from Sigma-Aldrich (Italy). The EO-NPs were prepared following Werdin González *et al*.^34^ with some modifications. In brief, PEG 6,000 (100 g) was melted at 65 °C on a hotplate stirrer. Then, 10 g of each essential oil were added to the melted PEG, while stirring the mixture using a T25 digital ULTRA-TURRAX® (IKA, Germany) for 30 min at 15,000 rpm. The mixture was then cooled at −4 °C for 2 h and completely ground in a refrigerated mortar. Finally, the product was sieved using a stainless steel sieve (230 mesh), stored at 25 ± 0.5 °C in an airtight container, and used for the bioassay within the following 48 h.

### EO-NP characterization

The EO-NP loading efficiency was calculated spectrophotometrically. Aliquots of PEG 6000 and EOs were diluted with absolute ethanol-water (3:1 v/v) and then stirred at 1000 rpm for 30 min. Serial dilutions were used to draw the standard curves. The absorbance of the solutions was determined by UV-visible analyses (Lambda 2SUV-vis spectrometer, Perkin Elmer) at the reference wavelengths of 313 nm for lemon, and 330 nm for both mandarin and orange, respectively.

A Dynamic Light Scattering (DLS) particle size analyser (NanoPartica SZ-100 apparatus, equipped with a 514 nm laser, Horiba Scientific) was used to assess the NPs surface charge at 25 °C, indicated by the zeta potential values, and the NP dimension, expressed in terms of Z-average size (d), and polydispersity index (PDI). After 24 h of EO-NP preparation, aliquots of each EO-NP were suspended in 10 mL of distilled water for 30 min and then the suspension was filtered using Whatman n° 1 filter paper^34^. The morphology of the EO-NPs was visualised using scanning electron microscope (Hitachi SU8020).

### Toxicological bioassays

All the experiments were conducted at the Department of Agriculture, Food and Environment of the University of Catania (Italy) under controlled environmental conditions in growth chambers (25 ± 2 °C, 60 ± 10% RH, 14:10 L:D). The EO and EO-NP solutions were prepared using an agitator for 15 minutes at 300 rpm for mixing: (i) the EOs or the EO-NPs at five different concentrations (2.5, 5, 10, 20 and 40 mg × mL^−1^), (ii) the same proportion of TWEEN 80 as emulsifier, and (iii) water.

The commercial insecticide indoxacarb (Steward^®^, DuPont™) was used as a positive control, because of its known efficacy in controlling the target pest. It was applied at the highest application rate recommended for tomato crops (12.5 g/hL). Water and TWEEN 80 + water were used as untreated controls. In preliminary trials, PEG particles alone were tested and no difference with the negative controls (both for insects and plants) were highlighted (data not shown). Therefore, this treatment was not included in the data analysis. Five replications per treatment were performed. The pest control efficacy of the tested compounds was evaluated by contact on eggs and larvae, and by ingestion on larvae.

### Contact Toxicity on Eggs (CTE)

Bioassays were carried out using 40 cm high tomato plants (40-d old, grown from seeds), exposed to *T. absoluta* adults for oviposition, as described above. These plants were then sprayed with the formulations, until run off, using a 2 L power-pack aerosol hand sprayer (Dea^®^, Volpi, Italy).

After drying for one hour, 10 treated eggs per replication were carefully transferred on a fine paintbrush to the untreated tomato shoots (with four expanded leaves). The shoots were placed inside a bioassay isolator made of 600 mL plastic glass. To prevent the shoots from dehydrating, each isolator was provided with a three cm layer of agar gel (15 g/L) in which the stem of the shoots was inserted. A fine mesh net was fixed on the upper opening of the glass to facilitate ventilation^60^.

### Translaminar Toxicity on Larvae (TTL)

In this experiment, 10 healthy second instar larvae per replication were transferred to untreated shoots. Larvae were left to settle until they entered the leaves by digging mines (about 2 h), after which they were sprayed, dried and isolated as described above.

### Ingestion Toxicity on Larvae (ITL)

In the ITL trial, tomato plants were sprayed and left to dry. Five shoots per treatment were collected and individually placed in the isolator described above. Ten second instar larvae per replicate were transferred to each treated shoot.

In all the trials, mortality was checked, using a binocular (at 12–36 magnifications), 24 and 72 h after the treatment. Eggs were considered dead when they became opaque, necrotic and/or appeared dehydrated. Larval mortality was assessed by stimulating the insects with a fine paintbrush, considering them dead if they remained immobile. In addition, the chronic toxicity of the tested compounds was assessed by calculating the proportion of juveniles, alive 72 h after the treatments, that reached the adult stage. Thus, 14 and 12 days after the egg and larvae exposure to the chemicals, respectively, the isolators were checked daily to record adult emergence.

### Phytotoxicity assessment

To evaluate the toxic effects of the tested formulations on the plants, five additional plants were sprayed for each treatment. Sprayed plants were kept in insect-proof cages in a greenhouse (min < *mean temperature* < max: 15.1 °C < 25.4 °C < 35.2 °C; min < *mean RH* < max: 36% < *62.9*% < 89%; natural ambient light in March-April). These were observed at 1, 3, 7 and 14 days after the treatments, recording the proportion of damaged leaves and damage severity. The damage severity was classified as: 0 (no damage), 1 (partially damaged leaf surface, with chlorosis and without necroses), 2 (leaves with evident necroses), 3 (dead leaves).

### Data analysis

The efficacy of the tested formulations was corrected for control mortality using Abbott’s formula^61^. Dependent variables were subjected to Levene and Shapiro-Wilk tests in order to assess the homogeneity and normality of variance across the groups, respectively, and transformed whenever needed. Repeated-measures analysis was conducted with the EO formulation (i.e., EO emulsion, EOs-NP) as the main effect, application rate as the covariate, and insect mortality registered at three different time intervals (i.e. 24, 72 h and at adult emergence) as the response variable. In addition, following the GLM procedure, within the datasets of each sampling time (24, 72 h and at adult emergence), a univariate analysis of variance was carried out with insect mortality as the dependent variable and the insecticide formulation (i.e. EOs emulsion, EOs-NP) and the application rate as fixed factors. Multiple comparisons were carried out using Duncan’s multiple range post-hoc test. Probit analysis was performed in order to estimate the median lethal concentrations (LCs_50_). Values were considered significantly different if their 95% fiducial limits did not overlap.

The phytotoxicity index was subjected to univariate analysis of variance, with formulations and time after the treatment as fixed factors and application rates as the covariate. Statistics were carried out using SPSS^®^ V. 20 (IBM). The phytotoxicity index (P_*i*_) was calculated as follows:

1 $${P}_{i}=\sum _{j=0}^{n}(\frac{DLj}{TL}\times \frac{DC}{n-1})$$

where *DL* is the number of damaged leaves for each damage severity class *j*, *TL* is the total number of leaves sprayed, *DC* is the damage severity class, and *n* is the number of damage severity classes. The *P*_*i*_ ranges from 0 (no damage) to 1 (dead leaves).

## Electronic supplementary material

Supplementary material

## References

[1] Desneux N, Luna MG, Guillemaud T, Urbaneja A (2011). The invasive South American tomato pinworm, *Tuta absoluta*, continues to spread in Afro-Eurasia and beyond: the new threat to tomato world production. J. Pest Sci..

[2] Tropea Garzia G, Siscaro G, Biondi A, Zappalà L (2012). *Tuta absoluta*, a South American pest of tomato now in the EPPO region: biology, distribution and damage. EPPO Bull..

[3] Haddi K (2012). Identification of mutations associated with pyrethroid resistance in the voltage-gated sodium channel of the tomato leaf miner (*Tuta absoluta*). Insect Biochem. Mol. Biol..

[4] Roditakis E (2017). Ryanodine receptor point mutations confer diamide insecticide resistance in tomato leafminer, *Tuta absoluta* (Lepidoptera: Gelechiidae). Insect Biochem. Mol. Biol..

[5] Abbes K (2015). Combined Non-Target Effects of Insecticide and High Temperature on the Parasitoid *Bracon nigricans*. PLOS ONE.

[6] Biondi A (2012). The non-target impact of spinosyns on beneficial arthropods. Pest Manag. Sci..

[7] Biondi A, Zappalà L, Stark JD, Desneux N (2013). Do Biopesticides Affect the Demographic Traits of a Parasitoid Wasp and Its Biocontrol Services through Sublethal Effects?. PLOS ONE.

[8] Sohrabi, F., Nooryazdan, H., Gharati, B. & Saeidi, Z. Evaluation of ten tomato cultivars for resistance against tomato leaf miner, *Tuta absoluta* (Meyrick) (Lepidoptera: Gelechiidae) under field infestation conditions. *Entomol. Gen*. 163–175, 10.1127/entomologia/2016/0350 (2016).

[9] Cocco A, Deliperi S, Delrio G (2013). Control of *Tuta absoluta* (Meyrick) (Lepidoptera: Gelechiidae) in greenhouse tomato crops using the mating disruption technique. J. Appl. Entomol..

[10] Zappalà L (2012). Efficacy of sulphur on *Tuta absoluta* and its side effects on the predator *Nesidiocoris tenuis*. J. Appl. Entomol..

[11] Zappalà L (2013). Natural enemies of the South American moth, *Tuta absoluta*, in Europe, North Africa and Middle East, and their potential use in pest control strategies. J. Pest Sci..

[12] Biondi A (2016). Can alternative host plant and prey affect phytophagy and biological control by the zoophytophagous mirid *Nesidiocoris tenuis*?. BioControl.

[13] Perdikis, D. & Arvaniti, K. Nymphal development on plant vs. leaf with and without prey for two omnivorous predators: *Nesidiocoris tenuis* (Reuter, 1895) (Hemiptera: Miridae) and *Dicyphus errans* (Wolff, 1804) (Hemiptera: Miridae). *Entomol. Gen*. 297–306, 10.1127/entomologia/2016/0219 (2016).

[14] Salehi, Z., Yarahmadi, F., Rasekh, A. & Sohani, N. Z. Functional responses of *Orius albidipennis* Reuter (Hemiptera, Anthocoridae) to *Tuta absoluta* Meyrick (Lepidoptera, Gelechiidae) on two tomato cultivars with different leaf morphological characteristics. *Entomol. Gen*. 127–136, 10.1127/entomologia/2016/0339 (2016).

[15] Isman MB (2006). Botanical Insecticides, Deterrents, and Repellents in Modern Agriculture and an Increasingly Regulated World. Annu. Rev. Entomol..

[16] Isman MB (2015). A renaissance for botanical insecticides?. Pest Manag. Sci..

[17] Pavela R, Benelli G (2016). Essential Oils as Ecofriendly Biopesticides? Challenges and Constraints. Trends Plant Sci..

[18] Miresmailli S, Isman MB (2014). Botanical insecticides inspired by plant–herbivore chemical interactions. Trends Plant Sci..

[19] Kumrungsee N, Pluempanupat W, Koul O, Bullangpoti V (2014). Toxicity of essential oil compounds against diamondback moth, *Plutella xylostella*, and their impact on detoxification enzyme activities. J. Pest Sci..

[20] Tak J-H, Jovel E, Isman MB (2015). Contact, fumigant, and cytotoxic activities of thyme and lemongrass essential oils against larvae and an ovarian cell line of the cabbage looper. Trichoplusia ni. J. Pest Sci..

[21] Abdelgaleil SAM, Mohamed MIE, Shawir MS, Abou-Taleb HK (2015). Chemical composition, insecticidal and biochemical effects of essential oils of different plant species from Northern Egypt on the rice weevil, *Sitophilus oryzae* L. J. Pest Sci..

[22] Campolo O (2016). Larvicidal Effects of Four Citrus Peel Essential Oils Against the Arbovirus Vector *Aedes albopictus* (Diptera: Culicidae). J. Econ. Entomol..

[23] Fekri, M. S., Samih, M. A., Imani, S. & Zarabi, M. The combined effect of some plant extracts and pesticide Pymetrozine and two tomato varieties on biological characteristics of *Bemisia tabaci* (Homoptera: Aleyrodidae) in greenhouse conditions. *Entomol. Gen*. 229–242, 10.1127/entomologia/2016/0020 (2016).

[24] Campolo O (2014). Fumigant bioactivity of five Citrus essential oils against *Tribolium confusum*. Phytoparasitica.

[25] Krčmar, S. & Gvozdić, V. Field studies of the efficacy of some commercially available essential oils against horse flies (Diptera: Tabanidae). *Entomol. Gen*. 97–105, 10.1127/entomologia/2016/0121 (2016).

[26] Conti B, Canale A, Cioni PL, Flamini G, Rifici A (2011). *Hyptis suaveolens* and *Hyptis spicigera* (Lamiaceae) essential oils: qualitative analysis, contact toxicity and repellent activity against *Sitophilus granarius* (L.) (Coleoptera: Dryophthoridae). J. Pest Sci..

[27] Tomé HVV, Cordeiro EMG, Rosado JF, Guedes RNC (2012). Egg exposure to pyriproxyfen in the tomato leaf miner *Tuta absoluta*: ovicidal activity or behavioural-modulated hatching mortality?. Ann. Appl. Biol..

[28] Romeo FV, Luca SD, Piscopo A, Poiana M (2008). Antimicrobial Effect of Some Essential Oils. J. Essent. Oil Res..

[29] Campolo O (2014). Effects of inert dusts applied alone and in combination with sweet orange essential oil against *Rhyzopertha dominica* (Coleoptera: Bostrichidae) and wheat microbial population. Ind. Crops Prod..

[30] Cardiet G, Fuzeau B, Barreau C, Fleurat-Lessard F (2011). Contact and fumigant toxicity of some essential oil constituents against a grain insect pest *Sitophilus oryzae* and two fungi, *Aspergillus westerdijkiae* and *Fusarium graminearum*. J. Pest Sci..

[31] Moretti MDL, Sanna-Passino G, Demontis S, Bazzoni E (2002). Essential oil formulations useful as a new tool for insect pest control. AAPS PharmSciTech.

[32] Sasson, Y., Levy-Ruso, G., Toledano, O. & Ishaaya, P. D. I. In *In*sectici*de*s Design *Using Advanced Technologies* (eds Ishaaya, P. D. I., Horowitz, P. D. A. R. & Nauen, D. R.) 1–39, 10.1007/978-3-540-46907-0_1 (Springer Berlin Heidelberg, 2007).

[33] Kah M, Beulke S, Tiede K, Hofmann T (2013). Nanopesticides: State of Knowledge, Environmental Fate, and Exposure Modeling. Crit. Rev. Environ. Sci. Technol..

[34] Werdin González JO, Gutiérrez MM, Ferrero AA, Fernández Band B (2014). Essential oils nanoformulations for stored-product pest control - characterization and biological properties. Chemosphere.

[35] Mineo P (2017). Gold nanoparticles functionalized with PEGylate uncharged porphyrins. Dyes Pigments.

[36] Moreno SC, Carvalho GA, Picanço MC, Morais EG, Pereira RM (2012). Bioactivity of compounds from *Acmella oleracea* against *Tuta absoluta* (Meyrick) (Lepidoptera: Gelechiidae) and selectivity to two non-target species. Pest Manag. Sci..

[37] de Oliveira JL, Campos EVR, Bakshi M, Abhilash PC, Fraceto LF (2014). Application of nanotechnology for the encapsulation of botanical insecticides for sustainable agriculture: Prospects and promises. Biotechnol. Adv..

[38] Gogos A, Knauer K, Bucheli TD (2012). Nanomaterials in Plant Protection and Fertilization: Current State, Foreseen Applications, and Research Priorities. J. Agric. Food Chem..

[39] Perlatti, B., *et al*. In *Insecticides - Development of Safer and More Effective Technologies* (ed. Trdan, S.) (InTech, 2013).

[40] Müller RH, Jacobs C, Kayser O (2001). Nanosuspensions as particulate drug formulations in therapy. Adv. Drug Deliv. Rev..

[41] Regnault-Roger C, Vincent C, Arnason JT (2012). Essential Oils in Insect Control: Low-Risk Products in a High-Stakes World. Annu. Rev. Entomol..

[42] Melliou E (2009). High quality bergamot oil from Greece: Chemical analysis using chiral gas chromatography and larvicidal activity against the West Nile virus vector. Mol. Basel Switz..

[43] Michaelakis A (2009). Citrus essential oils and four enantiomeric pinenes against *Culex pipiens* (Diptera: Culicidae). Parasitol. Res..

[44] Suwansirisilp K (2012). Behavioral responses of *Aedes aegypti* and *Culex quinquefasciatus* (Diptera: Culicidae) to four essential oils in Thailand. J. Pest Sci..

[45] Song J-E, Kim J-M, Lee N-H, Yang J-Y, Lee H-S (2016). Acaricidal and Insecticidal Activities of Essential Oils against a Stored-Food Mite and Stored-Grain Insects. J. Food Prot..

[46] Koppel AL, Herbert DA, Kuhar TP, Malone S, Arrington M (2011). Efficacy of Selected Insecticides Against Eggs of *Euschistus servus* and *Acrosternum hilare* (Hemiptera: Pentatomidae) and the Egg Parasitoid *Telenomus podisi* (Hymenoptera: Scelionidae). J. Econ. Entomol..

[47] Beament JWL (1952). The Role of Cuticle and Egg-Shell Membranes in the Penetration of Insecticides. Ann. Appl. Biol..

[48] Margulis-Goshen, K. & Magdassi, S. In *Advanced Technologies for Managing Insect Pests* (eds Ishaaya, I., Palli, S. R. & Horowitz, A. R.) 295–314, 10.1007/978-94-007-4497-4_15 (Springer Netherlands, 2013).

[49] Lee S-E (2001). Fumigant toxicity of volatile natural products from Korean spices and medicinal plants towards the rice weevil, *Sitophilus oryzae* (L). Pest Manag. Sci..

[50] Tripathi AK, Prajapati V, Khanuja SPS, Kumar S (2003). Effect of d-Limonene on Three Stored-Product Beetles. J. Econ. Entomol..

[51] Fang R (2010). Insecticidal activity of essential oil of *Carum Carvi* fruits from China and its main components against two grain storage insects. Mol. Basel Switz..

[52] Yang K (2014). Bioactivity of essential oil of Litsea cubeba from China and its main compounds against two stored product insects. J. Asia-Pac. Entomol..

[53] Malacrinò A, Campolo O, Laudani F, Palmeri V (2016). Fumigant and Repellent Activity of Limonene Enantiomers Against *Tribolium confusum* du Val. Neotrop. Entomol..

[54] Hollingsworth RG (2005). Limonene, a Citrus Extract, for Control of Mealybugs and Scale Insects. J. Econ. Entomol..

[55] Wittstock U, Gershenzon J (2002). Constitutive plant toxins and their role in defense against herbivores and pathogens. Curr. Opin. Plant Biol..

[56] Akhtar, Y. & Isman, M. B. In *Advanced Technologies for Managing Insect Pests* (eds Ishaaya, I., Palli, S. R. & Horowitz, A. R.) 231–247, 10.1007/978-94-007-4497-4_11 (Springer Netherlands, 2013).

[57] Kimbaris AC, Papachristos DP, Michaelakis A, Martinou AF, Polissiou MG (2010). Toxicity of plant essential oil vapours to aphid pests and their coccinellid predators. Biocontrol Sci. Technol..

[58] Synowiec, A., Kalemba, D., Drozdek, E. & Bocianowski, J. Phytotoxic potential of essential oils from temperate climate plants against the germination of selected weeds and crops. *J. Pest Sci*. 1–13, 10.1007/s10340-016-0759-2 (2016).

[59] Poonpaiboonpipat, T. *et al*. Phytotoxic effects of essential oil from *Cymbopogon citratus* and its physiological mechanisms on barnyardgrass (*Echinochloa crus-galli*). *Ind. Crops Prod*. **41**, 403–407 (2013).

[60] Biondi A, Desneux N, Siscaro G, Zappalà L (2012). Using organic-certified rather than synthetic pesticides may not be safer for biological control agents: Selectivity and side effects of 14 pesticides on the predator *Orius laevigatus*. Chemosphere.

[61] Abbott WS (1987). A method of computing the effectiveness of an insecticide. J. Am. Mosq. Control Assoc..

