# Author Correction: Citrus peel essential oil nanoformulations to control the tomato borer, *Tuta absoluta*: chemical properties and biological activity

**DOI:** 10.1038/s41598-018-28931-8

**Published:** 2018-07-13

**Authors:** Orlando Campolo, Asma Cherif, Michele Ricupero, Gaetano Siscaro, Kaouthar Grissa-Lebdi, Agatino Russo, Lorena M. Cucci, Patrizia Di Pietro, Cristina Satriano, Nicolas Desneux, Antonio Biondi, Lucia Zappalà, Vincenzo Palmeri

**Affiliations:** 1 University of Reggio Calabria, Dipartimento di AGRARIA, Loc. Feo di Vito, 89122 Reggio Calabria, Italy; 2 University of Catania, Department of Agriculture, Food and Environment, via Santa Sofia 100, 95123 Catania, Italy; 3 University of Carthage, Laboratoire d’Entomologie-Acarologie, Institut National Agronomique de Tunisie, 43 Avenue Charles Nicolle, 1082 Cité Mahrajène, Tunis Tunisia; 4 University of Catania, Department of Chemical Sciences, Viale Andrea Doria 6, 95125 Catania, Italy; 5 INRA (French National Institute for Agricultural Research), Université Nice Sophia Antipolis, CNRS, UMR 1355-7254, Institut Sophia Agrobiotech, 06903 Sophia Antipolis, France

Correction to: *Scientific Reports* 10.1038/s41598-017-13413-0, published online 12 October 2017

The original version of this Article listed an incorrect Affiliation for Agatino Russo. The correct Affiliation is listed below:

University of Catania, Department of Agriculture, Food and Environment, via Santa Sofia 100, 95123, Catania, Italy.

This has now been corrected in the HTML and PDF versions of this Article, and in the accompanying Supplementary Information file.

