# Supplementary material for: Citrus peel essential oil nanoformulations to control the tomato borer, Tuta absoluta: chemical properties and biological activity
Source: Sci Rep. 2017 Oct 12;7:13036. doi: 10.1038/s41598-017-13413-0 (PMC5638903; doi:10.1038/s41598-017-13413-0)
Supplement: Supplementary file 1 — Supplementary material [file 41598_2017_13413_MOESM1_ESM.docx]

**Electronic Supplementary Material**

**Citrus peel essential oils nanoformulations to control the tomato borer, *Tuta absoluta*: chemical properties and biological activity**

**Orlando Campolo ^1^, Asma Cherif ^2,3^, Michele Ricupero ^2^, Gaetano Siscaro ^2^, Kaouthar Grissa-Lebdi^3^, Agatino Russo ^2^, Lorena M. Cucci ^4^, Patrizia Di Pietro ^4^, Cristina Satriano ^4^, Nicolas Desneux ^5^, Antonio Biondi ^2^, Lucia Zappalà ^2*^, Vincenzo Palmeri ^1^**

^1^ University of Reggio Calabria, Dipartimento di AGRARIA, Loc. Feo di Vito, 89122, Reggio Calabria, Italy

^2^ University of Catania, Department of Agriculture, Food and Environment, via Santa Sofia 100, 95123, Catania, Italy

^3^ University of Carthage, Laboratoire d’Entomologie-Acarologie, Institut National Agronomique de Tunisie, 43 Avenue Charles Nicolle, 1082 Cité Mahrajène, Tunis, Tunisia;

^4^ University of Catania,Department of Chemical Sciences, Viale Andrea Doria 6, 95125, Catania, Italy

^5^ INRA (French National Institute for Agricultural Research), Université Nice Sophia Antipolis, CNRS, UMR 1355-7254, Institut Sophia Agrobiotech, 06903 Sophia Antipolis, France

^*^ Corresponding author: lzappala@unict.it

**
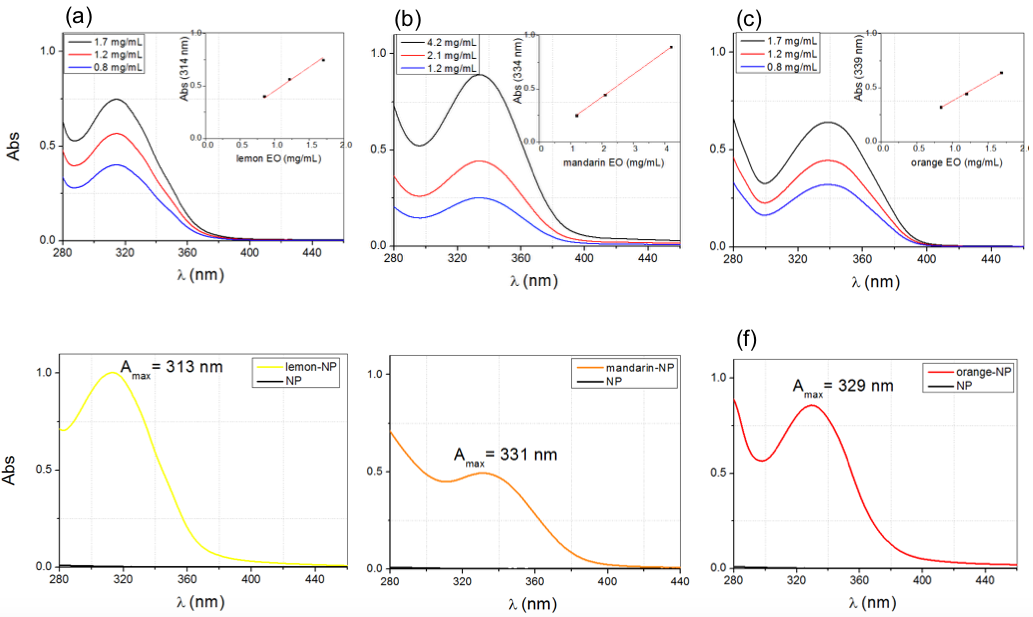
**

(d)

(e)

**Figure S1.** (a-c): Calibration curves obtained by UV-visible absorption spectra of EOs in ethanol/water (3:1, v/v): a) lemon (0.8 mg/mL, 1.2 mg/mL, 1.7 mg/mL); b) mandarin (1.2 mg/mL, 2.1 mg/mL, 4.2 mg/mL); c) orange (0.8 mg/mL, 1.2 mg/mL, 1.7 mg/mL). (d-f): UV-visible spectra of EO-NPs (25 mg/mL) in ethanol/water (3:1,v/v) compared with bare PEG-NP: d) lemon EO-NP; e) mandarin EO-NP; f) orange EO-NP.

| **Sample** | **EtOH/H_2_O (3:1, v/v)** | |
| --- | --- | --- |
|  | ε (mL·cm^-1^·mg^-1^) | Standard Error |
| lemon EO | 0.454 | ±0.005 |
| mandarin EO | 0.212 | ±0.001 |
| orange EO | 0.379 | ±0.003 |

**Table S1.** Molar extinction coefficients calculated from the linear fit of the UV-visible spectra showed in Fig.S1 a-c for the EOs dissolved in ethanol/water (3:1, v/v).
